# Modeling Fabric Movement for Future E-Textile Sensors

**DOI:** 10.3390/s20133735

**Published:** 2020-07-03

**Authors:** Roope Ketola, Vigyanshu Mishra, Asimina Kiourti

**Affiliations:** ElectroScience Laboratory, Dept. of Electrical and Computer Engineering, The Ohio State University, Columbus, OH 43212, USA; ketola.2@osu.edu (R.K.); mishra.186@osu.edu (V.M.)

**Keywords:** e-textiles, fabric movement, joint flexion, modeling, transmission coefficient, wearable motion capture, wearable sensors

## Abstract

Studies with e-textile sensors embedded in garments are typically performed on static and controlled phantom models that do not reflect the dynamic nature of wearables. Instead, our objective was to understand the noise e-textile sensors would experience during real-world scenarios. Three types of sleeves, made of loose, tight, and stretchy fabrics, were applied to a phantom arm, and the corresponding fabric movement was measured in three dimensions using physical markers and image-processing software. Our results showed that the stretchy fabrics allowed for the most consistent and predictable clothing-movement (average displacement of up to −2.3 ± 0.1 cm), followed by tight fabrics (up to −4.7 ± 0.2 cm), and loose fabrics (up to −3.6 ± 1.0 cm). In addition, the results demonstrated better performance of higher elasticity (average displacement of up to −2.3 ± 0.1 cm) over lower elasticity (average displacement of up to −3.8 ± 0.3 cm) stretchy fabrics. For a case study with an e-textile sensor that relies on wearable loops to monitor joint flexion, our modeling indicated errors as high as 65.7° for stretchy fabric with higher elasticity. The results from this study can (a) help quantify errors of e-textile sensors operating “in-the-wild,” (b) inform decisions regarding the optimal type of clothing-material used, and (c) ultimately empower studies on noise calibration for diverse e-textile sensing applications.

## 1. Introduction

Wearable sensors have long been reported for a variety of healthcare, sports, and other applications [1,2,3,4,5,6,7]. Traditionally, such wearable sensors rely on rigid copper for their implementation, resulting in bulky accessory-like devices that are worn on the body (e.g., smart watches, fitness trackers [1,2,3,4,5,6,7]). However, as we progress into the future, seamlessness and unobtrusiveness are strongly desired for wearable sensors [3]. To this end, flexible e-textile sensors that are embedded into fabrics are becoming increasingly popular. Over the years, example fabrication approaches for e-textile sensors have ranged from copper tape adhered on fabrics [8] to metallized fabrics [9], conductive inks [10], and, more recently, conductive e-threads [11].

A major limitation of e-textile sensors is that they are necessarily affected by the movement and positioning of the garments used. That is, sensors can shift and move along with the body as a result of garment movement, resulting in error (or, equivalently, noise). Unfortunately, this noise has not been accounted for in the area of e-textile sensing. Notably, the majority of works have considered the validation upon static and controlled phantom models that do not reflect the dynamic nature of wearables. For example, the authors of [12] proposed an e-textile sensor to monitor the accumulation of lung fluid, and all tests were conducted using a static torso phantom. In another case [13], a wearable sensor was reported for monitoring joint flexion and rotation, and all tests were conducted using a static limb model and with the sensor tacked into grooves to avoid any potential drift. Clearly, these studies are far from emulating real-world scenarios.

Literature has recognized that garments move/drift, but when it comes to e-textiles, unrealistic (symmetric) crumpling models have been employed. For example, the coplanar wearable waveguide antenna described by the authors of [14] showed an excessive detuning of ~2 GHz under a symmetrical crumpling pattern caused by bending the elbow. In another case [15], simulations with symmetric deformation concluded that “crumpling can have a serious effect on the resonant frequency, bandwidth and radiation from textile antennas.” That is, realistic models for fabric movement have not been developed for e-textiles. To overcome the above, the authors of [16] recommended placing e-textiles on flat areas of the human body so that the effects of crumpling are reduced. Expectedly, such applications may not always be possible. In summary, it is important to measure and quantify garment motion in order to understand and eliminate related errors for future e-textile sensors. 

To model garment motion, currently available methods utilize fixed model-based cloth simulations, data-driven approaches, and 3D video/imaging methods (see Table 1). Cloth simulations use time-intensive particle-based physics simulations [17] or finite-element methods [18,19,20], which break the cloth into a structure of small geometric shapes. Simulation methods for modelling cloth motion are common technology but are typically poorly applicable to wearable sensors and e-textiles. The simulations are not perfectly realistic because they make various assumptions about the physical qualities of the material, and often do not take into account external forces (such as wind or gravity), or mechanical properties of the cloth, such as the individual threads, stretching, and friction [19]. In addition, the algorithms take a long time to run—specifically, the resolution of the collisions of simulation elements is time-consuming [21]. Data-driven approaches rely on building and training complicated models, which require the collection of data and take time to train. Hence, these are only applicable when used with similar type of data that serve to train the models, and are thus not easily generalizable [22,23]. Even more importantly, the vast majority of the aforementioned works have been targeted toward computer animation applications, and it is unclear whether and how the resulting models could be adapted to e-textile sensors. Methods that involve 3D scans, whether with video or with images, typically require up to dozens of cameras. These cameras must be calibrated and synchronized together, and often utilize specialized software [24,25,26,27]. In addition, the computational time of these methods is high: The authors of [27] cited 16 seconds of computation per frame when using video. However, the accuracy of 3D imaging is very high [28], and a lot of positional data is acquired without necessitating the use of markers. This makes 3D imaging generalizable. But, even with markers, the logistics of 3D imaging methods are complicated. For example, the authors of [29] used animatronic mannequins, infrared (IR) motion capture systems, and several cameras positioned around the mannequin in order to track garment motion. Hence, although 3D video/imaging can provide better accuracy, the end system is more complicated and requires higher logistics that lead to increased cost and computational time that are often unnecessary.

With the above in mind, this paper aimed to provide a simple, cost-effective, and general approach to quantify garment motion for use in e-textile sensor applications. Although studies on specific fabric properties have been performed with various methods [23,30], this paper presents the first experimental results for fabric movement in three general categories of fabrics: Loose, tight, and stretchy fabrics. All three types highlight the need to take fabric-related noise into account. As a case study, performance was contrasted for our previous joint flexion sensor [31], in the absence and presence of garment motion per fabric movement errors reported in this paper. The marker-based image processing approach used in this paper was designed to be cost-effective and to require minimal logistics for setup. Our ultimate aims were to (a) enable a simple yet useful methodology that accounts for realistic garment noise of e-textile sensors, (b) inform decisions on the type of selected clothing-material, and (c) empower future studies on noise calibration for e-textile sensors operating “in-the-wild.”

## 2. Materials and Methods

### 2.1. Phantom Model and Physical Markers

**Figure 1:**
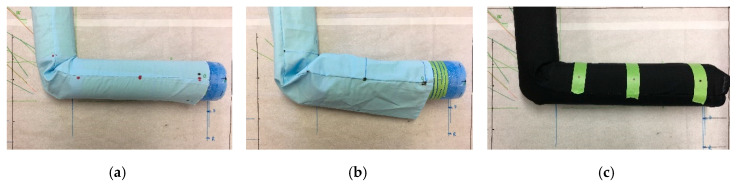
In vitro experimental setup of the three kinds of test sleeves, fitted around the Styrofoam arm: (**a**) Tight inelastic sleeve, (**b**) loose inelastic sleeve, and (**c**) stretchy sleeve.

Without loss of generality, our study focused on fabrics worn on the human arm and considered a canonical (cylindrical) arm model, capable of flexing in the 0° to 90^°^ range, as shown in Figure 1. Obviously, the approaches reported herewith are generalizable, implying that other setups may readily be employed per the designer’s needs, namely other parts of the human body, anatomical as opposed to canonical phantom models, other types of motion beyond flexion, narrower or wider range of motion, and so on. In addition, the location and number of the markers can be adjusted depending on the type and position of the sensor used.

Referring to Figure 1, an in vitro arm phantom was constructed, consisting of two cylinders of Styrofoam (permittivity, ε_r_ ~ 1, loss tangent, tanδ ~ 1), 4 cm in radius. As expected, the dielectric properties of the phantom were not of relevance to this particular study. The cylinders were attached with a goniometer at the joint to emulate human limb extension and flexion, similar to [20]. Thereafter, three test sleeves were sewn together around the arm, individually corresponding to three experiments. These include: (a) A tight sleeve of inelastic cotton fabric (see Figure 1a), (b) a loose sleeve of inelastic cotton fabric (see Figure 1b), and (c) a stretchy sleeve of Nylon Spandex fabric (80% Nylon, 20% Spandex; see Figure 1c). A fourth sleeve, a stretchy sleeve (95% Polyester, 5% Spandex) with lower elasticity, was constructed to compare stretchy sleeves of varying elasticity, with results presented in Section 3.4. Note that throughout the rest of this paper (except Section 3.4), the term stretchy sleeve refers only to the higher elastic (80% Nylon, 20% Spandex) stretchy sleeve shown in Figure 1c.

**Figure 2:**
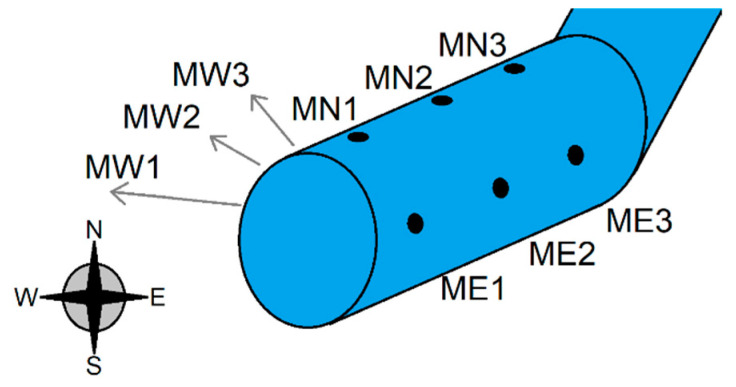
An example of the markers that were used to track the displacement of the sleeve. The markers were drawn onto the sleeves and labeled using cardinal directions with the convention MW1: “Marker West 1” and so forth. There was a total of nine markers on the sleeve, three on each side.

For each sleeve type, three markers were placed on three different sides of the sleeve—designated West (W), North (N), and East (E)—on the left, top, and right sides of the arm, for a total of nine markers around the sleeve (see Figure 2). Recording position and displacement of these markers enabled us to capture the sleeve’s movement in all three dimensions.

The selection of the number and location of these markers were done based on two factors: (a) Proof-of-concept demonstration of the capability of this method to capture three0dimensional fabric movement, and (b) in regard to specific type of sensor for which the fabric movement was captured. For (b), a wearable sensor [31] was considered, which was used in the case study discussed in Section 3.5. The sensor used in the case study was composed of coils wrapped around the arm, and it was affected by the motion of the coils along the forearm. Therefore, this sensor was sensitive to larger-scale shifts in the position of the garment relative to the arm. The markers utilized allow for the measurement of the relevant fabric displacement, so that the fabric displacement can be related to the noise in the sensor. If the designer uses a different sensor and is interested in different garment motion, the markers can be placed in new positions, and the relevant fabric motion can be measured. Additionally, more markers could be used to increase the resolution of the measured garment motion. However, the sensor discussed in Section 3.5 does not take up a wide area, hence the selection of the number and position of markers was sufficient.

### 2.2. Marker-Based Image Processing Approach

**Figure 3:**
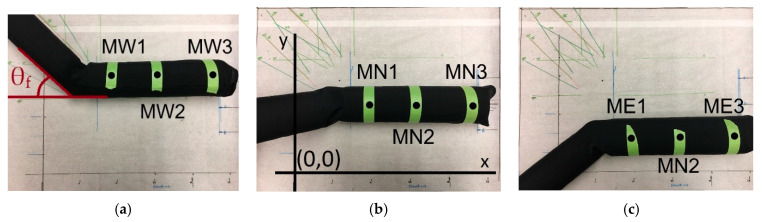
Sleeve sewn together on the arm and placed on plain background as captured by a fixed camera for (**a**) three MWs (Marker: West), (**b**) three MNs (Marker: North), and (**c**) three MEs (Marker: East). The sleeve shown here for demonstration is the stretchy sleeve. In addition, part (**a**) highlights the flexion angle (θ_f_), and (**b**), the coordinate axes and origin.

The sleeves of Figure 1 are placed on a plain background with a camera in a fixed location, as shown in Figure 3 for the example case of the stretchy sleeve. The background was marked with X and Y coordinate axes intersecting at the origin for all cases (shown explicitly in Figure 3b). The arm phantom and background were photographed, and the original position of the markers was captured by ImageJ (a Java-based image processing software for analyzing images) [32]. This original position was used as a reference position to compare the movement of the markers later. Using the bottom-left corner of the image (intersection of X and Y axes) as the origin and making sure that the arm and camera were lined up in the same way every time, the X and Y coordinates of each marker were recorded (Figure 3). The implication was that movement in the X direction referred to movement of the markers along the arm, toward and away from the elbow and hand, and movement in the Y direction referred to movement of the markers radially around the arm. Using simple marks on the background, a scale was established, and the coordinates were converted to centimeter units. Each photograph recorded three markers at a time, starting with the West side (Figure 3a), followed by rotation of the arm in the frame of the image to capture the other six markers on the North (Figure 3b) and East sides (Figure 3c). Each side of the arm therefore had a different reference position in the frame.

### 2.3. Experimental Approach and Data Collection

The abovementioned phantom was capable of performing extension/flexion quantified by flexion angle (θ_f_) (see Figure 3a). Extension/flexion was manually enforced on the phantom for the limb movement, as outlined below, and the corresponding displacement in the fabric was recorded with the help of the nine markers. Three sets of data were collected during these measurements. First, qualitative observations of the movements of the sleeves were recorded. Second, 10 consecutive flexion/extension cycles were performed (i.e., moving the arm from θ_f_ = 0° to 90° and back to 0° for 10 times) on each type of sleeve, and the displacement at the end of these cycles was recorded at the position of θ_f_ = 0° (extension). This provided information on movement of the sleeve over time. Third, the displacement of each marker was recorded with respect to flexion angle (θ_f_), for θ_f_ = 0°, 30°, 45°, 60°, and 90° (using the convention that θ_f_ = 0° corresponds to full extension, or, equivalently, straight arm). This provided information on the movement of the sleeve with respect to flexion angle. Finally, a comparison was conducted between two stretchy fabrics, as one had more elastic than the other. This comparison was also made by measuring the marker displacements after 10 consecutive flexion/extension cycles. This experiment was conducted to highlight the effect of varying elasticity in stretchy fabric.

## 3. Results

Qualitative and quantitative comparison is hereafter reported for all three sleeves (loose, tight, and stretchy), followed by comparison between stretchy sleeves of varying elasticities. The goal was to contrast the performance of each sleeve and streamline an approach that takes into account this noise in an e-textiles sensors context. To this end, and as a proof-of-concept, an e-textile joint flexion sensor was considered [31], and its performance was compared in the absence and presence of fabric movement.

### 3.1. Qualitative Observations

**Figure 4:**
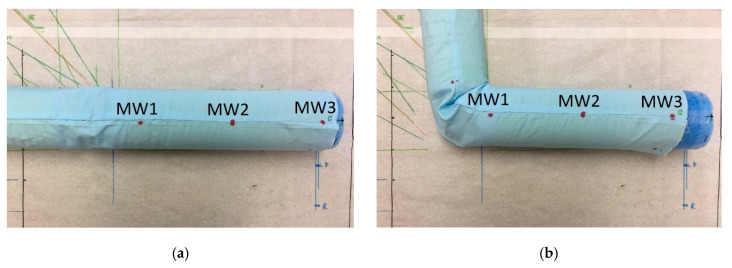
Experimental setup of arm with tight sleeve in (**a**) extension (at 0°) with no displacement and (**b**) after flexion (θ_f_ = 90°), showing the displacement of the sleeve caused by the fabric bunching up at the joint.

Qualitative observations of each type of sleeve indicated that the loose sleeve was moving much more freely and was largely affected by gravity, as expected. The measurements of the position of the tight and stretchy sleeves were not affected by gravity, because the sleeves were tight enough around the arm that friction kept them in place when the arm was turned around. Finally, a general trend was observed during flexion—the sleeve bunched up near the elbow, pulling the fabric toward the joint. This trend is depicted in Figure 4. Here, Figure 4a shows the original position of the garment and markers at full extension (θ_f_ = 0°), and Figure 4b shows the new position of the garment and markers after a 90° flexion of the arm. These displacements are quantified in Section 3.2. This bunching phenomenon was mostly prevalent in the tight sleeve, but it was also observed in the loose and stretchy sleeves, although to a lesser extent (as expected). This is also elaborated further in Section 3.2.

### 3.2. Quantitative Observations of Sleeve Motion over Time

The sleeves were compared over 10 consecutive flexions, as described in Section 2.2. The displacement for the three markers on each side was recorded in the X and Y directions. For each side, the average over all three markers is reported in Table 2. The uncertainty used in the displacements was the standard deviation of the measures of the X and Y displacements (per the coordinate system of Figure 3).

As seen, the markers on the sleeve tended to drift toward the joint: The average displacement in the X direction was −1.9 cm for the loose sleeve, −4.0 cm for the tight sleeve, and −2.1 cm for the stretchy sleeve. This average displacement of the markers for the loose and the stretchy sleeves were smaller in magnitude than for the tight sleeve. The poor performance of the tight sleeve can be explained by the bunching up of the sleeve, as displayed in Figure 4. The loose sleeve also bunches up similar to the tight sleeve but is free to fall back into place once the arm is straightened. The stretchy sleeve bunches up less than the tight sleeve because it can stretch across the arm. In the Y direction, the average displacements of the loose and tight sleeves were comparable at −1.3 cm and −1.6 cm, and the displacement of the stretchy sleeve was less than half of both, at 0.5 cm. 

The displacements of the loose sleeve and stretchy sleeve were comparable in the X direction, but the standard deviations of the displacements of the loose sleeve were higher than for the stretchy sleeve. The displacement of the stretchy sleeve was smaller in magnitude than for the loose sleeve for movement in the Y direction. This data supports the claim that the stretchy sleeve is the best-performing sleeve when the designer is concerned with the displacement of the fabric over time, as the arm moves. 

### 3.3. Quantitative Observations of Marker Diplacement with respect to Flexion Angle (θ_f_)

**Figure 5:**
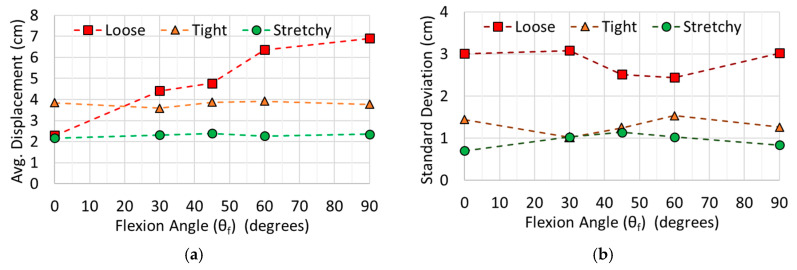
(**a**) Total average displacement, and (**b**) standard deviation (for all nine markers and in both X and Y directions) as a function of flexion angle (θ_f_) for all three types of sleeves.

Quantitative data on the displacement of the markers are shown in Figure 5a as a function of the flexion angle (θ_f_). Here, the X and Y displacement for each marker was used to find the magnitude of the total displacement, and the average for all of the markers on the West, North, and East sides was taken for all three sleeves. The average displacement and corresponding standard deviation for each sleeve is shown in Figure 5a,b, respectively. Note that these measurements were performed after each sleeve underwent 10 cycles of flexions, so the fabric had time to drift. Hence, the data in Figure 5 depended only on the flexion angle rather than the drift from the original position of the fabric. This is why there was an initial displacement at θ_f_ = 0° (Figure 5a).

As seen, the results confirm our qualitative observations. Figure 5 further demonstrates that the loose sleeve had highly varying average displacement (2.3–6.9 cm) across different θ_f_ and a very large standard deviation (±2.4–±3.1 cm). This is again consistent with the qualitative observations made in Section 3.1 (the loose sleeve was very likely to move about inconsistently), and the quantitative data from Table 2. However, this was not the case for tight and stretchy sleeves, which had quite consistent average displacement (tight: 3.6–3.9 cm, stretchy: 2.2–2.4 cm) and standard deviation (tight: ±1.0–±1.5 cm, stretchy: ±0.7–±1.1 cm).

Overall, results confirm that wearable sensors that require a consistent or predictable position relative to the body should utilize a tight-fitting and stretchy clothing rather than a loose one (which is more difficult to model). Nevertheless, the phenomenon displayed in Figure 4 and results of Table 2 should still be taken into account while making selection between tight and stretchy clothing.

### 3.4. Quantitative Comparison of Stretchy Sleeves with Varying Elasticities

In order to compare performance among different types of stretchy sleeves, the stretchy sleeves with higher elasticity (80% Nylon, 20% Spandex, same as used above) and lower elasticity (95% Polyester, 5% Spandex) were compared over 10 consecutive flexions, as described in Section 2.2. The displacement for the three markers on each side was recorded in the X and Y directions. For each side, the average over all three markers is reported in Table 3. The uncertainty used in the displacements was the standard deviation of the measures of the X and Y displacements (per the coordinate system of Figure 3). 

Table 3 clearly demonstrates that displacement in the stretchy sleeve with higher elasticity was smaller in magnitude for all of the data points except one (italicized). On average, the displacement in the X direction for the sleeve with a lower elasticity was −3.7 cm, compared to −2.1 cm for the sleeve with a higher elasticity. A stretchy sleeve with higher elasticity is capable of retaining its position without being pulled toward the areas where the fabric bunches up. This is consistent with the observations of the displacements of the stretchy sleeve and inelastic tight sleeve (elasticity ~ 0) shown in Table 2 in Section 3.2. In general, this data supports the conclusion that a sleeve that has more elasticity will shift less as a result of arm movement.

### 3.5. Case Study: E-Textile Sensor in the Absence/Presence of Fabric and Corresponding Drift

**Figure 6:**
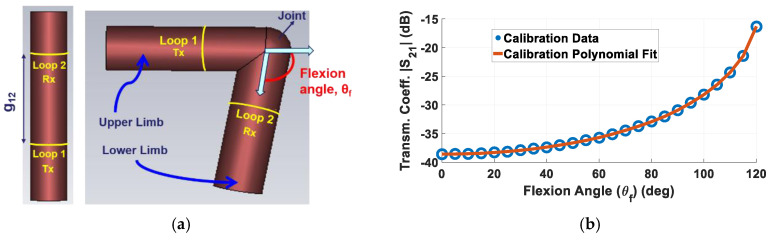
(**a**) Simulation setup for wearable loops to measure joint flexion angle [31]. This setup was used to obtain (**b**) calibration data (transmission coefficient |S_21_| vs. flexion angle (θ_f_)) for θ_f_=0° to 120° (at steps of 5°) along with corresponding polynomial fit serving as calibration file to predict θ_f_ from measured |S_21_| data.

To further understand the impact of fabric movement on e-textile sensor performance, we herewith consider an example case study of a wearable joint flexion sensor. Referring to Figure 6a, our recently reported sensor employs embroidered transmit (Tx) and receive (Rx) loops wrapped around the limb to reliably measure flexion angles. The operating principle relies on Faraday’s law of induction, as described in detail in [31]. Similar to Figure 1, the experimental setup in [31] involved a Styrofoam cylinder of 4 cm in radius to emulate the human arm, implying that the quantitative results of Section 3.2 and Section 3.3 are readily applicable to this case. 

Simulation setup corresponding to Figure 6a was created with loops placed symmetrically across the joint at g_12_ = 14 cm, which allowed a range of motion from θ_f_ = 0° to 120°. To generate calibration data for the sensor, simulations were performed (in the absence of fabric) using the finite integral technique-based CST^®^ simulator at a step size of 5° for θ_f_ = 0° to 120°, leading to total of 25 transmission coefficient (|S_21_|) data points. These |S_21_| results were then mapped to corresponding flexion angles using a polynomial fit obtained through Matlab^®^, as shown in Figure 6b. This serves as the calibration file and can be used to retrieve the flexion angle (θ_f_) corresponding to a certain measured transmission coefficient (|S_21_|) by the sensor.

In the presence of fabric movement, sensor loops will drift and |S_21_| measurements will be impacted accordingly. To demonstrate the effect on performance, a sensor embroidered on stretchy fabric (with higher elasticity) was assumed. To simulate the presence of the stretchy fabric, the average X-displacement corresponding to Figure 3 and the results of Figure 5a were incorporated in the setup of Figure 6a. This was achieved by shifting the loop based on the different fabric drifts at θ_f_ = 0° (−2.09 cm), 30° (−2.15 cm), 45° (−2.34 cm), 60° (−2.06 cm), and 90° (−2 cm). Note that Y-displacement was not considered, as the sensor’s performance was robust to changes around the arm due to symmetry [31]. 

**Figure 7:**
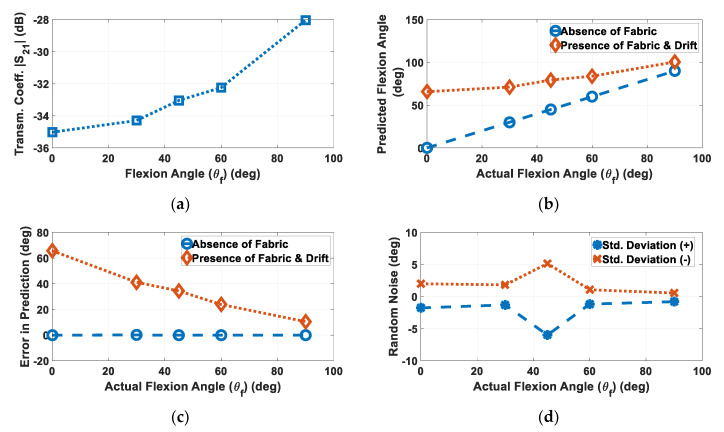
(**a**) Noisy |S_21_| data (due to fabric drift) obtained using simulation set-up of Figure 6a. (**b**) Predicted θ_f_ with respect to actual θ_f_ obtained via calibration plot of Figure 6b, both in the absence and presence of fabric drift, along with (**c**) respective error in predicted θ_f_, for both cases. High error outlines the importance of incorporating fabric drift model in the wearable sensors’ calibration. (**d**) Random noise caused during fabric drift vs. θ_f_, highlights the importance of specifying noise tolerance for wearable sensors.

Noisy |S_21_| data (impacted by drift) acquired by the sensor at the abovementioned angles are shown in Figure 7a. If we ignore fabric movement (as is the typical case with today’s e-textile sensors), then the data of Figure 7a would be mapped to sensor angles per the original calibration curve of Figure 6b. To this effect, predicted flexion angles and error caused in predicted flexion angles would be depicted by Figure 7b,c, respectively, both in the presence and absence of the fabric (and corresponding drift). Although the sensor is capable of retrieving the correct flexion angles in the absence of fabric drift, the error in prediction ranged from as high as 65.7° (at actual θ_f_ = 0°) to 10.5° (at actual θ_f_ = 90°) in the presence of fabric drift. This result clearly demonstrates that there can be significant impact on the sensor performance even for optimal fabrics that exhibit minimal drift, thereby delineating the importance of fabric drift modeling. Results also highlight the requirement of a separate calibration to be infused with the fabric drift model and predict the performance of such wearable sensors accurately, thereby calibrating noise. 

In addition, the standard deviation displayed in Figure 5b represents randomness in the drift itself, which would further add noise to the measurement. This standard deviation is incorporated on top of the drift in the simulation setup of Figure 6a, the results of which are summarized in Figure 7d. Noise in the range of −6°–5.1° was observed. This outlines the importance of, and specifies the noise tolerance for, such sensors as demonstrated for the sensor described by the authors of [13]. 

## 4. Discussion

The results demonstrate a method for quantifying and recording garment movement which enables fabric drift modeling. The setup which was utilized to demonstrate the method also allowed us to conduct a case study for a wearable loop sensor as described in Section 3.5, demonstrating, quantitatively, the importance of incorporating fabric drift modeling in the sensor. However, this method is not restricted to this setup or sensor and can be extended to any wearable sensor technology and any setup without the loss of generality. For instance, in the case of a square patch antenna, markers can be placed on the sleeve in the shape of a square in suitable locations for the antenna. Then, the method outlined in this paper could be used to track the movement of the parts of the garment that are relevant to that sensor in conjunction with antenna measurements.

In addition, this method can be refined using a more realistic arm phantom, or in vivo experiments on humans, in order to improve the accuracy and realism of the clothing motion. Experiments on humans could be conducted by photographing the subject in front of a background and measuring the garment motion corresponding to human motion. In addition, using more sophisticated software or programs to track the markers, and using video instead of only images, would allow for real-time comparisons, as well as measuring and tracking garment position simultaneously with wearable sensor measurements. Once the noise associated with fabric drift is modeled, it can be readily removed by infusing this information in the calibration file, thereby correcting the sensor performance in the presence of the fabric. This would enable sensor operation in practical settings and “in-the-wild.” For example, in the simulation case study presented in Section 3.5, the movement of the garment that was observed and displayed in Figure 5a resulted in measurement errors. The case study considered this fabric motion, and an actual flexion from 0° to 90° was mapped from 65.7° to 100.5° (Figure 7b) using calibration file (with unaccounted fabric drift) leading to significant error in prediction (Figure 7c). However, this error can be corrected by calibrating the noise using fabric drift model, which, in turn, can be developed through the marker-based method described in this work. 

Future research steps include (a) studying the motion of different fabrics to further inform clothing decisions when integrating sensors, and (b) taking measurements with sensors embroidered onto clothing and tracking the garment movement in real-time and dynamic settings. As suggested by the case study in Section 3.5, these would allow for a comparison of noisy sensor measurements to baseline measurements without a sleeve, and the noise caused by garment shifting could be calibrated and hence removed, leading to error-free measurements.

## 5. Conclusions

A simple and inexpensive marker-based image processing method was presented to model noise caused due to fabric drift, which can affect the performance of the wearable sensors. Three different types of sleeves were used for this study to cover different options generally used by humans, namely loose, tight, and stretchy. The suitable placement of nine markers in different directions allowed the capture of three-dimensional fabric movement. It was found that stretchy fabrics provided the most consistent and predictable motion. Particularly, tight and stretchy clothing was reported to have a significantly lower standard deviation (maximum of ±0.8 cm for tight and ±0.5 cm for stretchy) than loose clothing (maximum of 1.9 cm) as a result of being constrained closer to the body and having much more predictable movement. Additionally, the drift experienced by stretchy clothing over time was found to be comparable to loose clothing, but ~50% of that experienced by tight clothing. This suggests that stretchy sleeves had the best performance, since they had both the lowest drift and the lowest standard deviation. In addition, it was found that even within stretchy sleeves, a sleeve with higher elasticity performed better with respect to abovementioned parameters. A case study was then performed to successfully demonstrate the impact on sensor performance using stretchy fabric with higher elasticity. If the fabric drift modeling was ignored, an error as high as 65.7° was found. But, with availability of such fabric drift model, the drift noise impacting the performance of the sensor can be eliminated by informing the calibration file. This method can be easily modified and extended to various shapes and sizes of sensors which are applicable to different parts of the human body, thereby opening doors for modeling real-time fabric movement pertinent to diverse wearable sensing scenarios.

## Figures and Tables

**Table 1:** Comparison of state-of-the-art textile-modeling methods with respect to desirable parameters.

Methods	Low Complexity	Low Cost	Accurate ^1^	Generalizable	Low Computational Cost and Time
Simulations [21]	No(-)	No(-)	No (-)	No(-)	No(-)
Data Driven Models [22,23]	No(-)	No(-)	Yes(+)	No(-)	No(-)
3D Video/Imaging [24,25,26,27,28,29]	No(-)	No(-)	Yes(+)	Yes(+)	No(-)
Proposed Method	Yes(+)	Yes(+)	Yes(+)	Yes(+)	Yes(+)

Note: In this context, “Accurate” refers to sufficient accuracy for modelling the resultant noise in a sensor.

**Table 2:** Average displacements and standard deviations in the X and Y directions for the three markers on each side, for the loose, tight, and stretchy sleeves, at θ_f_ = 0° after 10 consecutive flexions. The last row also contains the averages and standard deviations of the data across the West, North, and East sides for comparison.

Markers	Loose Sleeve		Tight Sleeve		Stretchy Sleeve	
	Average X Displacement	Average Y Displacement	Average X Displacement	Average Y Displacement	Average X Displacement	Average Y Displacement
West	−1.6 ± 0.3 cm	−3.6 ± 1.0 cm	−3.9 ± 0.1 cm	−1.5 ± 0.2 cm	−2.3 ± 0.1 cm	0.5 ± 0.2 cm
North	−1.8 ± 0.1 cm	−1.3 ± 0.6 cm	−3.5 ± 0.1 cm	−2.6 ± 0.6 cm	−2.0 ± 0.1 cm	−0.1 ± 0.1 cm
East	−2.3 ± 0.2 cm	1.0 ± 0.6 cm	−4.7 ± 0.2 cm	−0.7 ± 0.2 cm	−2.2 ± 0.1 cm	1.0 ± 0.5 cm
Average	−1.9 ± 0.3 cm	−1.3 ± 1.9 cm	−4.0 ± 0.5 cm	−1.6 ± 0.8 cm	−2.1 ± 0.1 cm	0.5 ± 0.4 cm

**Table 3:** Average displacements and standard deviations in the X and Y directions for the three markers on each side, for the more stretchy and less stretchy sleeves, at θ_f_ = 0° after 10 consecutive flexions. The last row also contains the averages and standard deviations of the data across the West, North, and East sides, for comparison.

Markers	Stretchy Sleeve (Lower Elasticity)		Stretchy Sleeve (Higher Elasticity)	
	Average X Displacement	Average Y Displacement	Average X Displacement	Average Y Displacement
West	−3.8 ± 0.2 cm	0.9 ± 0.2 cm	−2.3 ± 0.1 cm	0.5 ± 0.2 cm
North	−3.8 ± 0.3 cm	−1.0 ± 0.1 cm	−2.0 ± 0.1 cm	−0.1 ± 0.1 cm
East	−3.6 ± 0.3 cm	*−0.4 ± 0.2 cm*	−2.2 ± 0.1 cm	1.0 ± 0.5 cm
Average	−3.7 ± 0.1 cm	−0.2 ± 1.0 cm	−2.1 ± 0.1 cm	0.5 ± 0.4 cm
